# The marine phosphorus cycle

**DOI:** 10.3389/fmicb.2013.00105

**Published:** 2013-05-21

**Authors:** Angelicque White, Sonya Dyhrman

**Affiliations:** ^1^College of Oceanography and Atmospheric Sciences, Oregon State UniversityCorvallis, OR, USA; ^2^Woods Hole Oceanographic InstituteWoods Hole, MA, USA

Phosphorus, the 13th element to be discovered on our planet, has a rich and varied history spanning match-making, bombs, fertilizer, and pesticides. Entire islands economies (Nauru) have collapsed in the mad hunt for phosphorus rock (Gowdy and McDaniel, 1999). Mineral reserves of this critical and valuable element are now being so rapidly mined from our Earth's crust that we are approaching a point where demand exceeds supply (Cordell et al., 2009). Why such a storied element? The answer rests in the fact that phosphorus is an essential ingredient in every known recipe for life: it is integral for energy storage, cell structure, and the very genetic material that encodes all life on the planet. Phosphorus is in fact, the staff of life (Karl, 2000), the scaffolding on which all biomass is built. Just as phosphorus fertilizer supports the growth of agricultural crops, phosphorus supply supports the growth of photosynthetic organisms, or phytoplankton, at the base of the marine food web. The biogeochemical cycling of phosphorus in the ocean is thus an important determinant of ecosystem structure and function, which motivated this special research topic in *Frontiers in Microbiology* entitled “The marine phosphorus cycle.” In total, 10 papers have been included, with 8 original research articles and 2 reviews. Collectively these papers detail new advances, important nuances, and unexpected discoveries related to phosphorus cycling in the marine environment.

This e-book begins with a number of papers detailing the production of dissolved organic phosphorus (DOP) in the upper water column. Original research by Mackey et al. (2012) documents the rapid uptake of phosphate by harmful algal bloom forming diatom species in Monterey Bay and the subsequent production of DOP as these blooms decayed. This latter finding is relatively novel- simply because relatively little is known about the rate and control of DOP production in the sea. To address this point, Ruttenberg and Dyhrman (2012) show that changes in the ambient inorganic nitrogen:phosphorus ratio may modulate the rate of DOP production in coastal upwelling regimes. Taken together these papers provide new insights into how DOP is produced in the ocean.

Roughly balancing this production is biotic decomposition, which occurs as microbes scavenge organic pools for essential elements. For DOP compounds characterized by a C-O-P bond (esters), hydrolysis is catalyzed by a class of enzymes termed alkaline phosphatases. Organisms with the capacity to build this enzyme can hydrolyze DOP to access phosphorus, and if heterotrophic, carbon as well. Work by Lin et al. (2012) has shown that alkaline phosphatases are widely distributed within a certain class of marine plankton termed dinoflagellates. The twist is that the enzyme is differentially located within the cell in different lineages. This suggests that organisms may have an organic niche, i.e., they have evolved strategies to utilize alkaline phosphatase in different ways so as to compete for select resources. In related work in the oligotrophic North Pacific, where phosphorus is sparingly low, Björkman et al. (2012) use a radioisotope approach to show that heterotrophic bacteria and the ubiquitous photoautotroph *Prochlorococcus* are equal competitors for phosphate whereas heterotrophic bacteria are much more effective at scavenging model DOP compounds. These papers illuminate the myriad of mechanisms and strategies used by microbes to access organic resources.

Heterotrophic organisms may not always hydrolyze DOP to acquire phosphorus for growth; rather these organisms may decompose DOP to access carbon and or nitrogen, leaving the cleaved phosphate in dissolved inorganic form—remineralized. The remineralization of phosphorus proceeds as a function of the availability of the resource, the elemental requirements of the remineralizing bacteria, the lability of organic phosphorus resources, and environmental controls such as temperature. These critical processes are highlighted in several papers in this e-book. Suzumura et al. (2012) show that DOP remineralization rates are higher in the high biomass coastal ocean than the open ocean, whereas DOP hydrolysis appears to be inversely related to phosphate concentrations in the open ocean. In effect, the liberation of phosphate from organic compounds is a function of the number of enzymatically-active bacterial and autotrophic cells as well as the relative availability of phosphate that suppresses the need to hydrolyze organic substrates. Scott et al. (2012) then show that different bacterial isolates (freshwater in this case) differentially regulate their phosphorus composition in response to the C:P ratio of the organic substrate. Increasing the phosphorus content of substrates, thereby decreasing C:P ratios, leads to luxury phosphorus uptake by some isolates and net phosphorus remineralization by others. In this sense, the rate of phosphorus remineralization will then depend on the stoichiometric demands and the diversity of bacterial populations. There are environmental pressures on DOP remineralization as well. White et al. (2012) report that temperature is predictably related to DOP decay constants for select individual substrates, albeit the slope of the relationship varies widely between substrates (e.g., a mono-ester versus a nucleotide) and with differing microbial communities. These findings suggest that the environmental control of labile DOP remineralization will not be easily captured with a single master equation. Adding to the complexity, all of the above work largely refers to enzymatic degradation of C-O-P linked organic matter. There are other bond classes that are important sources of phosphorus nutrition: specifically, the C-P linked phosphonate compounds which are reviewed by Villarreal-Chiu et al. (2012). The enzymes that hydrolyze these bonds are poorly understood relative to alkaline phosphatases, but are no less important in the remineralization of DOP. Collectively, these papers describe the nuances and complexity of DOP degradation in the ocean.

Moving from phosphorus cycling in the surface ocean to the action at depth, Flores et al. (2012) trace the contours of phosphorus in sediment records in the South Atlantic section of the Southern Ocean and find clues for a past “White Ocean”; a period of Earth's history when phosphorus scrubbed off continental margins during glacial highs fueled blooms of carbonate-shelled coccolithophores. These events would have enhanced the phosphorus burial rate in these rich polar ocean regimes. In addition to sinking of living and detrital cells, phosphorus also reaches the sediment adsorbed onto iron oxyhydroxides coating the surfaces of clay particles and colloids. Once buried, a complex series of biotic and abiotic processes lead to iron dissolution, release of phosphate and the precipitation of the phosphorus-rich mineral apatite. Extraction of these sedimentary phosphorite rocks fuels ~85% of the world's phosphate consumption. Yet the mechanisms by which this apatite forms are not entirely clear. Crosby and Bailey (2012) review the evidence for a microbial role in apatite formation; hypothesizing that fossilized bacteria found in phosphorite rock may be harbingers for the rapid precipitation of apatite by sulfide-oxidizing bacteria.

Even now, over 300 years after the discovery of phosphorus, we are still unraveling the mechanisms, rates, and controls of phosphorus cycling on our planet. This compilation presents a snapshot of the diverse interdisciplinary research at the leading edge of these continued efforts. These collective papers provide a greater understanding of the rapid and critical cycling of phosphorus in the surface ocean, of the mechanisms, specificities, and rates of decomposition of organically bound phosphorus, and of the formation and preservation of phosphorus in the sediment—they are an exciting step forward.
